# Hospital Medical Schools and the King's Fund

**Published:** 1910-11-26

**Authors:** 


### HOSPITAL MEDICAL SCHOOLS AND THE KING'S FUND.

### AN ACCUSATION AND A REPLY.

We have received the following correspondence, which we publish as received :—

To his Gracious Majesty King George V.

May it please your Majesty.

The Metropolitan Radical Federation, for whom I am empowered to write, have observed with deep concern that some of those who manage the great hospitals of London are persons who have not attended with becoming respect to the recommendations clearly and emphatically pronounced by Sir Edward Fry, his Grace the Archbishop of York, and Lord Welby in the report made by them in 1905 to the Council of King Edward's Hospital Fund after they had fully investigated the financial relations existing between the great hospitals and the schools contiguous to them.

Those three investigators, whose probity and wisdom none can gainsay, declared that the benefits reciprocally conferred by the hospitals and schools the one upon the other should be regarded as equivalent, and that therefore, where money was taken from the coffers of the hospital and conveyed to those of the school, the school remained a debtor to the hospital to the amount of such diversion.

To this declaration the Metropolitan Radical Federation appended its cordial adhesion when it was published. The schools are private enterprises which do not reveal their accounts to the public, and their profits, if any, are retained by private persons, and do not find their way into the treasury of the hospital.

This salutary direction that money subscribed or bequeathed to the hospitals, by persons whose sole interest is centred in the tending of the sick poor, should not be conveyed away to schools where the public are not informed what becomes of it has been openly flouted in one hospital and silently evaded in some others.

It has been openly flouted at St. George's, where Dr. Charles Slater, in a speech to the school, contemptuously spoke of the "ill-advised action of a commission of the King's Hospital Fund,"[1] and this insubordinate utterance is fully endorsed in the last published accounts of St. George's Hospital, where we discover[2] that £1,026 was paid directly from the funds of the hospital in salaries to the laboratory staff of the school, and £550[3] similarly diverted to the "Medical School for work done for the hospital," although the report of Sir Edward Fry and his distinguished coadjutors pronounced any "work done for a hospital" by a school as being paid for fully by the reciprocal benefit conferred upon the school by admission of its pupils to the wards of the hospital, and any cash

[1] *British Medical Journal*, October 3, 1908.
[2] Page 29 of Report for 1909. [3] Page 27.

payments such as these of £550 and £1,026 to be debts of the school to the hospital.

In these very clear circumstances the Metropolitan Radical Federation are disturbed, astonished, and grieved to observe that the Distribution Committee of King Edward's Hospital Fund have condoned this open flouting of the directions of their own distinguished committee of inquiry by making a grant to St. George's Hospital of £1,500, which appears in the same published accounts as the above insubordinate diversions.

In many other London hospitals vast sums in the past have been taken from the funds of the hospitals and conveyed into the private accounts of the schools; these amounts remain debts owing by the schools to the hospitals, but no effort appears as yet to have been made to repay them.

The Metropolitan Radical Federation make no apology for coming into the presence of your Majesty, who is President of King Edward's Hospital Fund, with the respectful request that your Majesty will at least direct that no further grant from King Edward's Hospital Fund be made to St. George's Hospital until the £1,576 diverted to the school, according to the last accounts, are refunded.

The Metropolitan Radical Federation further respectfully suggest that it would certainly give the working classes of London greater confidence in King Edward's Hospital Fund, and in its influence for strict finance at the hospitals, if its council were elected like the councils of other great philanthropic bodies, by the subscribers at an annual meeting, and not as at present without any reference to the subscribers.

The Metropolitan Radical Federation, for whom I am empowered to write this letter, represents over 40,000 working men of London; they therefore claim to speak more representatively for those for whom the hospitals were founded, exist, and are supported, than the Council of King Edward's Hospital Fund, who represent no one but themselves, and they confidently hope that your Majesty will not refer this letter to that Council, but will take it into your own gracious consideration, and direct that what we humbly ask for shall be done.—I am, your Gracious Majesty, Your obedient and humble servant,

37 Hall Street, (Signed) EDWARD GARRITY,
City Road, London, E.C. Honorary Secretary.

### THE REPLY.

KING EDWARD'S HOSPITAL FUND FOR LONDON,
7 Walbrook, E.C.,
Councillor E. Garrity, M.I.I., November 18, 1910.
Honorary Secretary,
Metropolitan Radical Federation.

SIR,—The communication addressed by you to his Majesty the King on the subject of the relations between hospitals and medical schools has been forwarded to the Fund by direction of the King, as his Majesty is now patron and no longer president of the Fund, and it has been considered by the Executive Committee.

The Committee feel sure that a knowledge of the facts will convince you that there is no ground whatever for the allegations against the Fund contained in your memorandum.

Your communication suggests that two departures from the findings of Sir Edward Fry's Commission of Inquiry have been committed by hospitals and condoned by this Fund. In the case of St. George's Hospital it is alleged that, contrary to the recommendations of the Commission, moneys were diverted in the year 1909 from the hospital to the school, and that the Fund by making a grant in that year condoned such diversions. It is also stated that in many other London hospitals vast sums have been conveyed in the past into the private accounts of the schools, and that no effort appears as yet to have been made to repay them.

In view of the reflections contained in your communication upon the alleged action of the King's Fund, we are directed to say, in the first place, that there is no ground for the suggestion that by making a grant to St. George's Hospital in 1909 the Fund has condoned any "flouting" of the directions of the Committee of Inquiry. A mere consideration of the date at which the grant was made will show that even if there has been any breach of the directions the Fund has not condoned it. The annual distribution takes place in December, and is necessarily based on the accounts of the preceding year, while in the case of hospitals with medical schools these accounts are supplemented by a certificate from the hospital that the accounts of the current year, when completed, will show that the rule as to medical schools has been observed. The accounts of St. George's Hospital, in which it is alleged that a breach of the directions is disclosed, were published some months after the last grant was made. The judgment of the Fund upon these accounts, whatever it may prove to be, will be expressed in the usual course by means of this year's distribution, and the time at which this judgment is formed and published has not yet arrived.

While it would be premature, therefore, for anyone connected with the Fund to express an opinion on the questions raised in reference to the accounts of St. George's Hospital, it may be added that the Fund was in correspondence with this hospital during the course of the year 1909 on the subject of the principles involved, and that the question whether or not these principles have been observed has necessarily come before the Fund, in the course of the ordinary examination of the accounts of the hospital with a view to the forthcoming distribution.

On the question of sums paid to medical schools prior to the date of the report of Sir Edward Fry's Commission of Inquiry, the action of the Fund is equally clear. The phrase in the report on which your charge is based, namely, "the schools still remain debtors to the hospitals in respect of these pecuniary contributions made to them" is qualified by a reference to a previous paragraph; and as a result of this qualification the Commission arrived at the conclusion that "the great uncertainty which, in many or all cases, attends any attempt to ascertain the intentions of the original founders of the hospitals and medical schools, the impossibility of knowing what were, from time to time, the exact motives in the minds of the subsequent donors and subscribers, and the complexity of the relations which have existed between hospitals and schools, seem to render any attempt to open up the transactions in the past between the hospitals and schools very undesirable, even if it were possible." The attitude of the Fund towards these past payments is based upon the actual terms of the report of the Commission.

The Fund is administered under the provisions of the Act of Parliament specially passed in 1907, and the governing body, appointed in accordance with that Act, may be relied upon to take whatever precautions may be necessary to ensure that the conditions which are binding on all hospitals applying for grants, including the condition that no part of the general funds subscribed for the relief of the sick poor is diverted to the purposes of medical education, are duly observed by every hospital which receives assistance from the Fund.—Yours faithfully,

(Signed) SAVILE CROSSLEY,
FREDERICK M. FRY,
Honorary Secretaries.

